# Quantifying mechanical and metabolic interdependence between speed and propulsive force during walking

**DOI:** 10.3389/fspor.2022.942498

**Published:** 2022-09-09

**Authors:** Richard E. Pimentel, Jordan N. Feldman, Michael D. Lewek, Jason R. Franz

**Affiliations:** ^1^Applied Biomechanics Laboratory, Joint Department of BME, UNC, and NCSU, University of North Carolina at Chapel Hill, Chapel Hill, NC, United States; ^2^Human Movement Science Laboratory, Division of Physical Therapy, University of North Carolina at Chapel Hill, Chapel Hill, NC, United States

**Keywords:** gait, locomotion, treadmill, walking economy, energy expenditure, push-off

## Abstract

Walking speed is a useful surrogate for health status across the population. Walking speed appears to be governed in part by interlimb coordination between propulsive (F_P_) and braking (F_B_) forces generated during step-to-step transitions and is simultaneously optimized to minimize metabolic cost. Of those forces, F_P_ generated during push-off has received significantly more attention as a contributor to walking performance. Our goal was to first establish empirical relations between F_P_ and walking speed and then to quantify their effects on metabolic cost in young adults. To specifically address any link between F_P_ and walking speed, we used a self-paced treadmill controller and real-time biofeedback to independently prescribe walking speed or F_P_ across a range of condition intensities. Walking with larger and smaller F_P_ led to instinctively faster and slower walking speeds, respectively, with ~80% of variance in walking speed explained by F_P_. We also found that comparable changes in either F_P_ or walking speed elicited predictable and relatively uniform changes in metabolic cost, together explaining ~53% of the variance in net metabolic power and ~14% of the variance in cost of transport. These results provide empirical data in support of an interdependent relation between F_P_ and walking speed, building confidence that interventions designed to increase F_P_ will translate to improved walking speed. Repeating this protocol in other populations may identify other relations that could inform the time course of gait decline due to age and disease.

## Introduction

Walking speed serves as a simple surrogate for human health status. For example, faster walking speeds associate with numerous health factors, including increased muscle strength, better cognitive function, greater independence, and reduced healthcare costs (Morris and Hardman, 1997; McGinn et al., 2008; Fritz and Lusardi, 2009; Dorsch et al., 2012; Stegemöller et al., 2014; Grau-Pellicer et al., 2019; Rasmussen et al., 2019). By understanding the mechanistic pathways contributing to slower walking speeds, we may identify avenues to maintain and restore independence and pedestrianism for safe and effective recreation, transport, and health in our population.

Biomechanically, walking speed is regulated in part by the magnitude of the peak anterior component of the ground reaction force - namely, the peak propulsive force (F_P_) (Hsiao et al., 2016). During push-off, the trailing leg generates vertical and horizontal forces that accelerate and redirect the body's center of mass (CoM) forward and upward. Simultaneously, trailing leg forces are opposed by braking forces (F_B_) which, in combination, facilitate smooth transitions from one step to the next (Donelan et al., 2002a). During steady-speed walking, F_P_ and F_B_ are relatively balanced during the step-to-step transition and their interaction explains much of the variance in mechanical work and metabolic cost required to walk (Donelan et al., 2002b; Kuo et al., 2005). Although interlimb coordination between F_P_ and F_B_ is what ultimately drives horizontal acceleration of the body's CoM, F_P_ has garnered disproportionately significant attention as a contributor to walking performance (Peterson et al., 2011; Hsiao et al., 2016; Browne and Franz, 2018; Hedrick et al., 2021; Herrero et al., 2021) and a target metric for intervention (Bowden et al., 2006; Hsiao et al., 2015a; Lewek et al., 2018; Liu et al., 2021). However, that attention is informed to date by observational studies unable to establish dependency and computational studies unable to establish whether model predictions manifest in human subjects. There is currently a lack of empirical data available to fully understand the independent effects of modifying F_P_ on walking speed and vice-versa.

Humans typically generate F_P_ *via* ankle plantarflexion using a combination of well-timed calf muscle contraction, elastic energy returned from the Achilles tendon, and effective limb orientation for mechanical advantage (Sawicki et al., 2009; Hsiao et al., 2015a; Lewek and Sawicki, 2019; Hedrick et al., 2021; Herrero et al., 2021). Because the ankle plantarflexor muscles and tendons account for ~60% of the work performed in typical gait (DeVita et al., 2007; Sawicki et al., 2009), it is no anomaly that plantarflexor pathologies affect both walking speed and walking economy (Schrack et al., 2016; Das Gupta et al., 2019; Tavakkoli Oskouei et al., 2021). This suggests that F_P_, walking speed, and metabolic cost are inextricably linked, posing a longstanding scientific challenge with significant potential for improved clinical countermeasures.

We often attribute the selection of walking speed to the minimization of metabolic cost. The cost of transport (CoT, i.e., net metabolic cost per unit distance traveled) during walking is *U*-shaped, with increasing costs as walking speed deviates from preferred. This suggests that our movement biomechanics and underlying muscle actions are tuned to minimize metabolic cost at our preferred speeds. Unfortunately, compared to young adults or unimpaired controls, numerous walking studies in older adults or people with gait limitations document higher CoT (Mian et al., 2006; Ortega and Farley, 2007; Jones et al., 2009; Schrack et al., 2016; Das Gupta et al., 2019) and slower preferred walking speeds (Jones et al., 2009; Schrack et al., 2016). Multiple factors likely explain the higher CoT in these individuals, including systemic factors (e.g., reduced cardiopulmonary function), local muscle and tendon factors (e.g., reduced muscle metabolic efficiency, lower tendon stiffness), and altered neural control or gait biomechanics (e.g., wider steps, increased co-activation, redistributing mechanical work to more proximal leg joints/muscles). However, the often-simultaneous presentation of slower speeds and higher CoT challenges our ability to fully understand the time-course of gait decline from aging or gait pathology.

Before scientists and clinicians can design and implement strategies to improve walking speed and lower metabolic cost in older adults or in individuals with gait pathology, we need to better understand exactly how F_P_ impacts walking speed in the context of metabolic cost. To our knowledge, no study has established empirical relations between F_P_, walking speed, and metabolic cost, even in unimpaired young adults. Thus, we designed a “clamp” protocol to meet this need by separately prescribing walking at a certain speed vs. walking with a certain F_P_. Our purpose was to: (1) determine whether increasing/decreasing F_P_ governs the selection of walking speed, and (2) quantify how the selection of F_P_ or walking speed impacts walking economy. Exploring these relations may build confidence that restoring F_P_ may improve walking speed. Additionally, our results may be useful when designing interventions or devices that improve walking ability and economy.

## Materials and methods

### Participants

Twenty young adults provided informed consent prior to participating in study activities. The University of North Carolina at Chapel Hill IRB approved all procedures. Participants were free of lower extremity injuries, neuromuscular complications, and assistive devices that might prevent protocol completion. Average (±standard deviation) demographics follow: 24.7 ± 5.2 years, 1.77 ± 0.11 m tall, 75.6 ± 13.7 kg, and BMI of 24.0 ± 3.4 kg/m^2^.

### Self-pace mode and biofeedback

**Figure 1:**
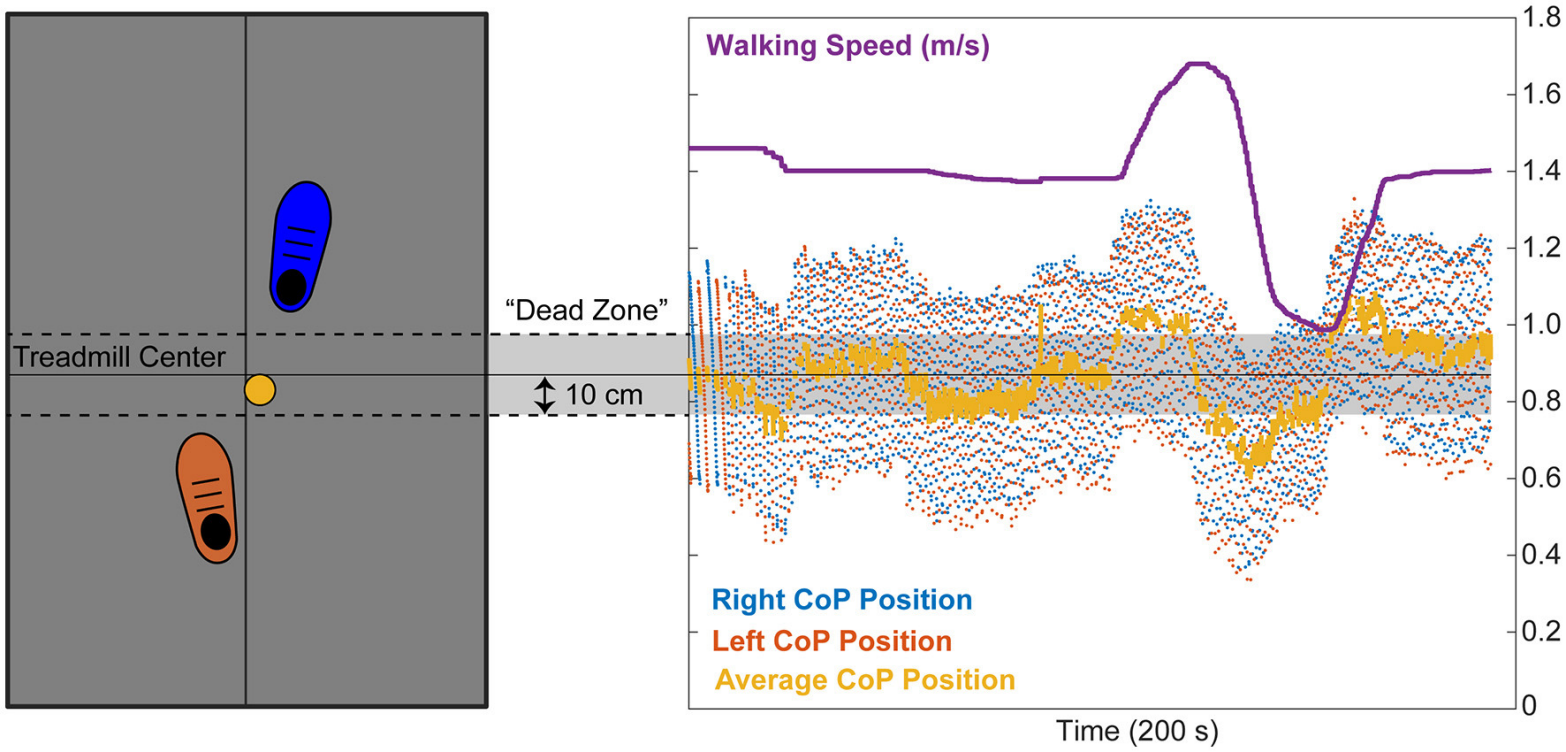
In self-pace mode, participants started walking on a split-belt treadmill at their preferred overground walking speed. We recorded participants' instantaneous bilateral centers of pressure (CoP) from each belt in real time and averaged the sides to estimate their relative fore/aft position on the treadmill (yellow dot and line). When the participant stayed centered on the treadmill (i.e., average CoP location during double support within the 20 cm “dead zone”), treadmill speed remained constant. When the participant's average CoP location during double support moved anterior or posterior to the dead zone, the treadmill speed increased or decreased linearly with the distance from center, respectively.

Understanding our design depends on first understanding our self-pace treadmill controller and targeted biofeedback. This study leveraged a self-paced treadmill controller adapted from Hedrick et al. (2021). Self-paced trials always started at the participant's preferred overground walking speed (see below), following which they could increase/decrease treadmill speed at will by moving forward/backward on the treadmill, respectively (Figure 1). We used participants' average center of pressure position during each double support phase to determine their relative anterior-posterior location on our force-sensing treadmill (Bertec Corp., Columbus, Ohio, USA). When the average center of pressure position moved outside the 20 cm “dead zone” centered on the treadmill midline, walking speed changed linearly with the distance from center:


$$\text{Δ}Speed=R^{*}D^{*}L\;$$

where R was the relative sign of speed change (−1 when posterior, and +1 when anterior to the dead zone), D defined the average center of pressure distance from treadmill center, and L linearly scaled the speed change (0.1 based on pilot testing).

In some trials, we also used real-time visual biofeedback to display the average peak F_P_ from the previous two steps (one from each side) on a screen in front of the participant with a target line representing the prescribed F_P_ according to our study protocol (see Protocol). We instructed participants to “match their push-off force to the target”. The biofeedback line turned green when participants' F_P_ was within 5% of the target value (in newtons), but was otherwise red. For additional encouragement, we provided a counter displaying the number of consecutive steps on target. We provide the Matlab-based (Mathworks, Natick, MA, USA) treadmill controller scripts at: https://www.github.com/peruvianox/FpBiofeedbackSelfPace.

### Protocol

We determined participants' preferred walking speed *via* the average from four 30-m hallway passes following instructions to “walk normally, as if down a sidewalk”. Participants completed a 3-min warm-up at their preferred walking speed followed by a 3-min familiarization with the self-pace treadmill mode and targeted F_P_ biofeedback. During those familiarizations, we ensured participants could increase/decrease F_P_ on command and regulate walking speed at will using self-pacing.

**Figure 2:**
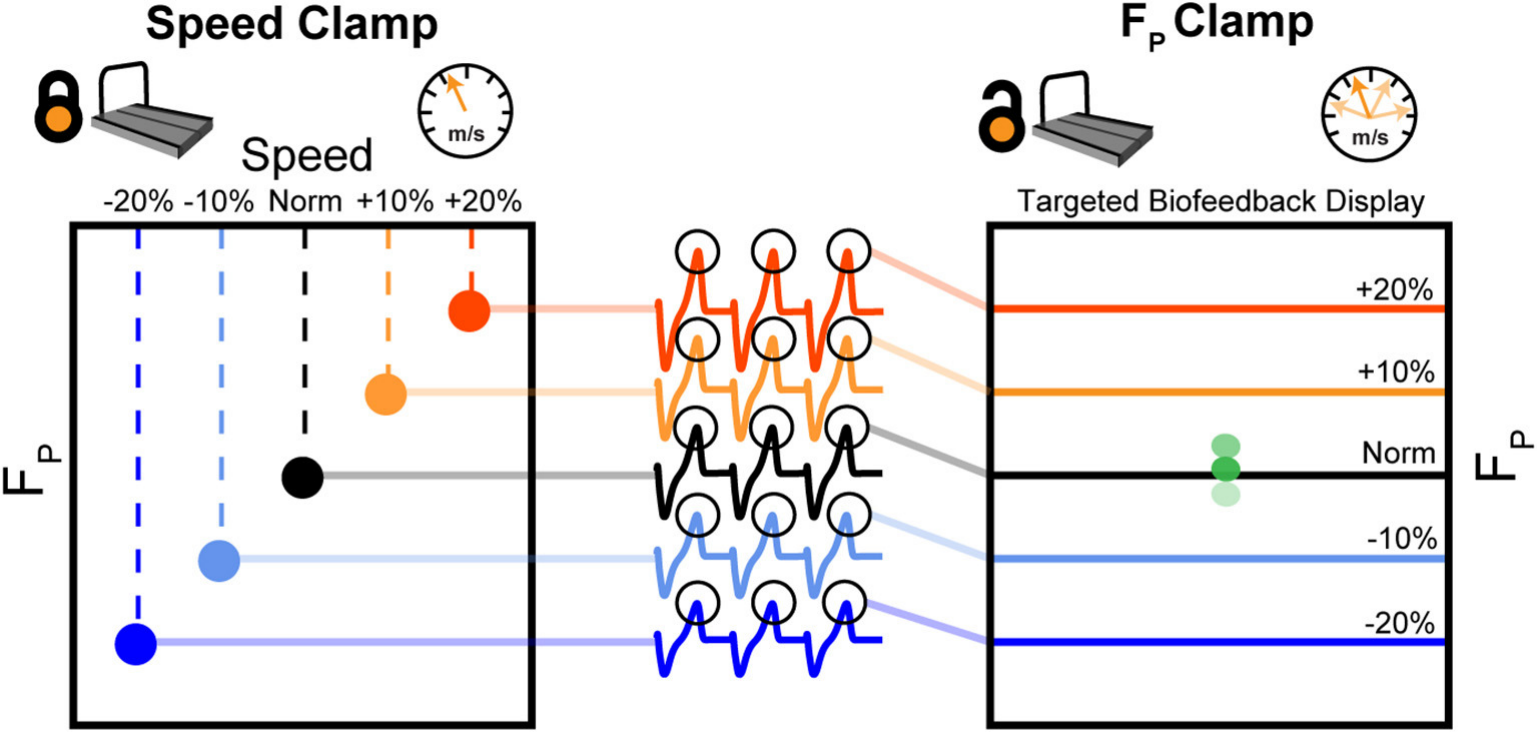
First, participants walked at their typical, overground walking speed (Norm) as well as ±10 and ±20% of Norm. During these 5-min, fixed-speed trials (speed clamp), we measured and averaged F_P_ from each step over the duration of the trial. During another set of five 5-min trials, we used targeted biofeedback and the self-paced treadmill mode to prescribe F_P_ by asking participants to target each of their average F_P_ values from the speed clamp while allowing participants to naturally adjust their walking speed (F_P_ clamp).

Figure 2 summarizes our protocol. Participants walked at a fixed speed (speed clamp) for five 5-min trials at their preferred speed (Norm) and at −20, −10, +10, and +20% of Norm in randomized order. We extracted the average peak F_P_ from each speed clamp trial to use as targets for the ensuing biofeedback trials. Participants then completed a randomized series of 5-min walking trials with biofeedback to target their average peak F_P_ from each speed clamp trial. These targeted biofeedback trials used self-pacing, thus prescribing a target F_P_ while allowing walking speed to vary (F_P_ clamp). Participants rested in a seated position for at least 1 min between each of the 10 trials, and could take longer rests *ad libitum* (average time between trials: 167 ± 105 s).

We recorded treadmill speed and ground reaction forces from the real-time treadmill controller script. The real-time interface between Cortex (Motion Analysis Corporation, Rohnert Park, CA, USA) and Matlab received data packets every 0.050 ± 0.002 s, with 10 embedded analog force samples at 1,000 Hz included in each packet. To match standardized methods for averaging expired gases during steady state walking (Gottschall and Kram, 2003; Griffin et al., 2003; Ortega and Farley, 2007; Peterson and Martin, 2010; Zukowski et al., 2017), we analyzed average speed and bilateral average peak F_P_ over the final 2 min of each trial for statistical analysis, allowing participants to explore and stabilize their walking patterns for the first 3 min of each trial. We measured braking force (F_B_) in post-processing by identifying the maximum posteriorly-oriented horizontal ground reaction force occurring during the first half of stance phase.

We also recorded walking kinematics *via* a 16-camera 3D motion capture system (Motion Analysis Corp., Rohnert Park, CA, USA) and retroreflective markers placed on anatomical landmarks of the lower limb. We do not report any joint kinematics in this article, yet we did use the locations of the heel markers at heel strike (when vertical ground reaction force surpasses 20 N) and toe off (when vertical ground reaction force drops below 20 N) instances to measure stride length and duration.

### Metabolic measurements

In a baseline standing trial and all walking trials, we sampled breath-by-breath exhaled oxygen and carbon dioxide using a COSMED K5 indirect calorimetry system (COSMED, Rome, Italy). To estimate standing and walking net metabolic power, we, respectively, averaged expired air measurements over the final 2 min of each collection. Standard regression equations estimated whole-body metabolic power from rates of oxygen consumption and carbon dioxide production (Brockway, 1987). We subtracted standing values from walking metabolic power to calculate net metabolic power, and lastly, normalized by body mass.

### Data analysis

We opted to normalize to preferred overground walking speed and habitual biomechanics (%body weight, W/kg, etc.) to align motor capacity and self-selected walking speed for each participant. We report squared Pearson correlations (*R*^2^) to quantify the relative variance explained between F_P_, walking speed, and metabolic outcomes (i.e., net metabolic power and CoT). We considered *R*^2^ strengths using the following classification (>0.8 = very strong, 0.6–0.8 = strong, 0.4–0.6 = moderate, 0.2–0.4 = weak, <0.2 = very weak). We used a two-way repeated measures analysis of variance (ANOVA) to identify main effects of and interactions between clamp type (speed vs. F_P_) and condition intensity (Norm, ±10%, ±20%) on walking speed, F_P_, net metabolic power, and CoT. When we found a significant main effect or interaction, we used Tukey's *post-hoc* tests to identify pairwise differences. We provide effect sizes for all significant statistical outcomes, with partial eta squared (ηp^2^) for ANOVA main effects and Cohen's *d* for *post-hoc* comparisons. We previously found statistically significant effects of altered F_P_ in measured (ηp^2^ = 0.58; Pieper et al., 2021) and model-predicted (ηp^2^ = 0.35; Pimentel et al., 2021) metabolic cost in an earlier cohort of *n* = 12 subjects. We increased the sample size to *n* = 20 to account for the novel self-pacing paradigm. With our sample, *post-hoc* analyses showed that we had >99% power for main effects of F_P_, speed, metabolic power, and CoT across our repeated measures ANOVAs for condition and clamp type. We performed all statistical processing in python using the Pingouin package (Vallat, 2018). For transparency, we provide our data and code at: https://github.com/peruvianox/SpeedFpClamp.

## Results

### Experimental performance

**Figure 3:**
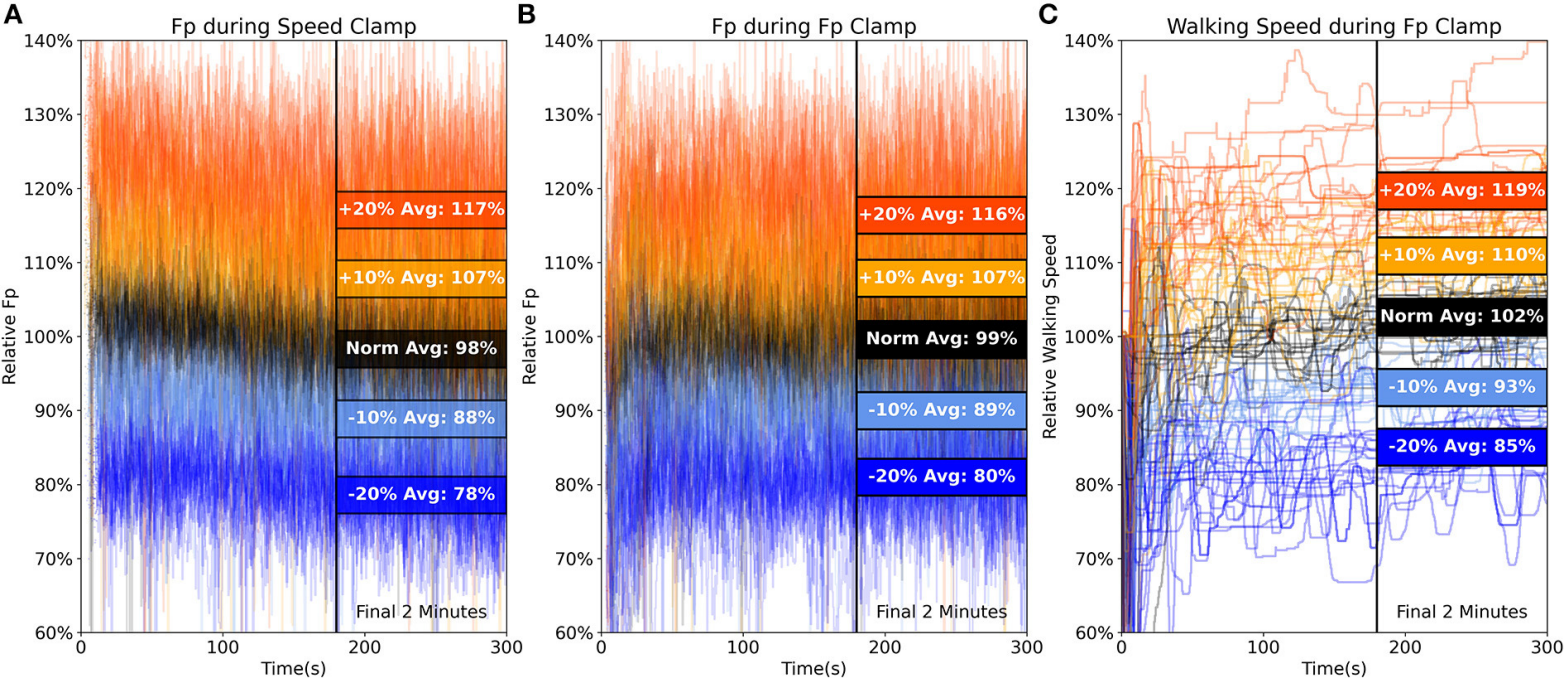
Here, we show a high-level visualization of the key outcomes from our study over the duration of each experimental trial. Averaged across every step (including left and right sides) during all trials and across all subjects, we show **(A)** relative F_P_ during the speed clamp, **(B)** F_P_ biofeedback targeting performance during the F_P_ clamp, and **(C)** relative instantaneous self-paced walking speed during the F_P_ clamp. In all panels, we also show the group-averaged outcomes over the final 2 min in colored blocks for each condition intensity. Overall, participant's average F_P_ and walking speed over the final 2 min generally agreed well with prescribed changes in condition intensity. Early variation in relative walking speed stabilized after the first ~60 s.

On average, participants walked at typical overground speeds of 1.41 ± 0.09 m/s and propelled themselves forward during treadmill walking with a typical F_P_ of 22.0 ± 2.3% body weight (*mean* ± *standard deviation*). Figure 3 shows: (A) F_P_ during speed clamp trials, (B) F_P_ clamp trial biofeedback targeting performance, and (C) subsequent changes in walking speed during F_P_ clamp trials. These panels demonstrate that participants modified F_P_ according to prescribed targets with accompanying changes in walking speed.

### Associations with walking speed

**Figure 4:**
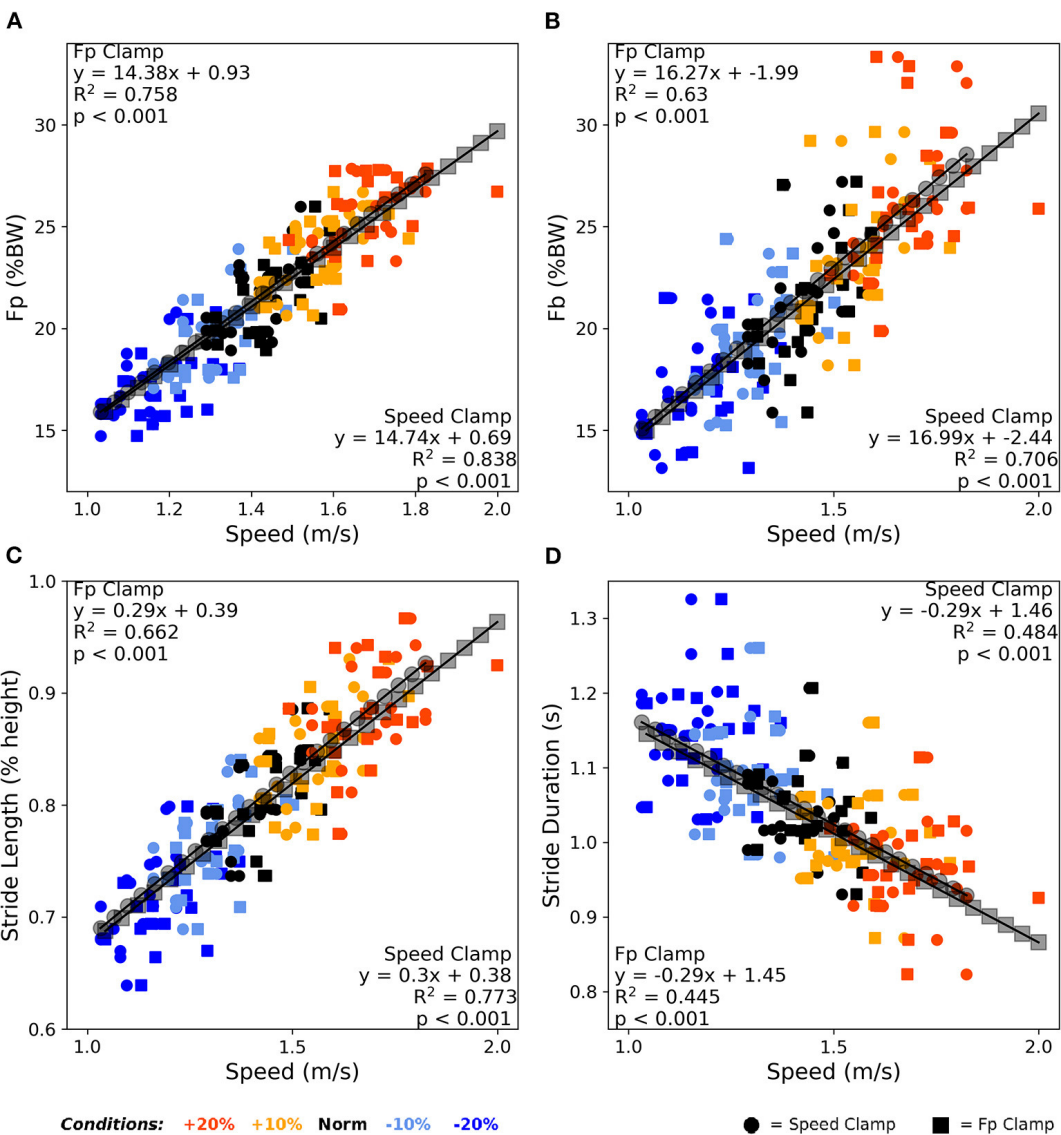
Averaged over the final 2 min, walking speed correlated very strongly with F_P_ **(A)**, strongly with F_B_ **(B)** and stride length **(C)**, and moderately with stride duration **(D)**. Participants responded nearly identically between clamp types across these four outcome variables to alter their walking speed over the condition intensities. BW, body weight.

Figure 4 shows that F_P_, F_B_, and stride kinematics all significantly correlated with walking speed (all *p* < 0.001). Reviewing the correlation strength and trendline similarity, F_P_ strongly associated with walking speed (average *R*^2^ = 0.80, Figure 4A), with similar slope coefficients (14.74 vs. 14.37) and variance explained (0.84 vs. 0.76) between the speed clamp and F_P_ clamp, respectively. F_B_ also strongly associated with walking speed (average *R*^2^ = 0.67, Figure 4B), with similar slope coefficients (16.99 vs. 16.27) and variance explained (0.63 vs. 0.71) between the clamp types. Finally, stride length and stride duration (Figures 4C,D) also correlated with walking speed (average *R*^2^ = 0.72 and 0.46, respectively) and exhibited very similar linear slope coefficients between clamp types (length: 0.29 vs. 0.3 and duration: −0.29 vs. −0.29).

### Associations with walking economy

**Figure 5:**
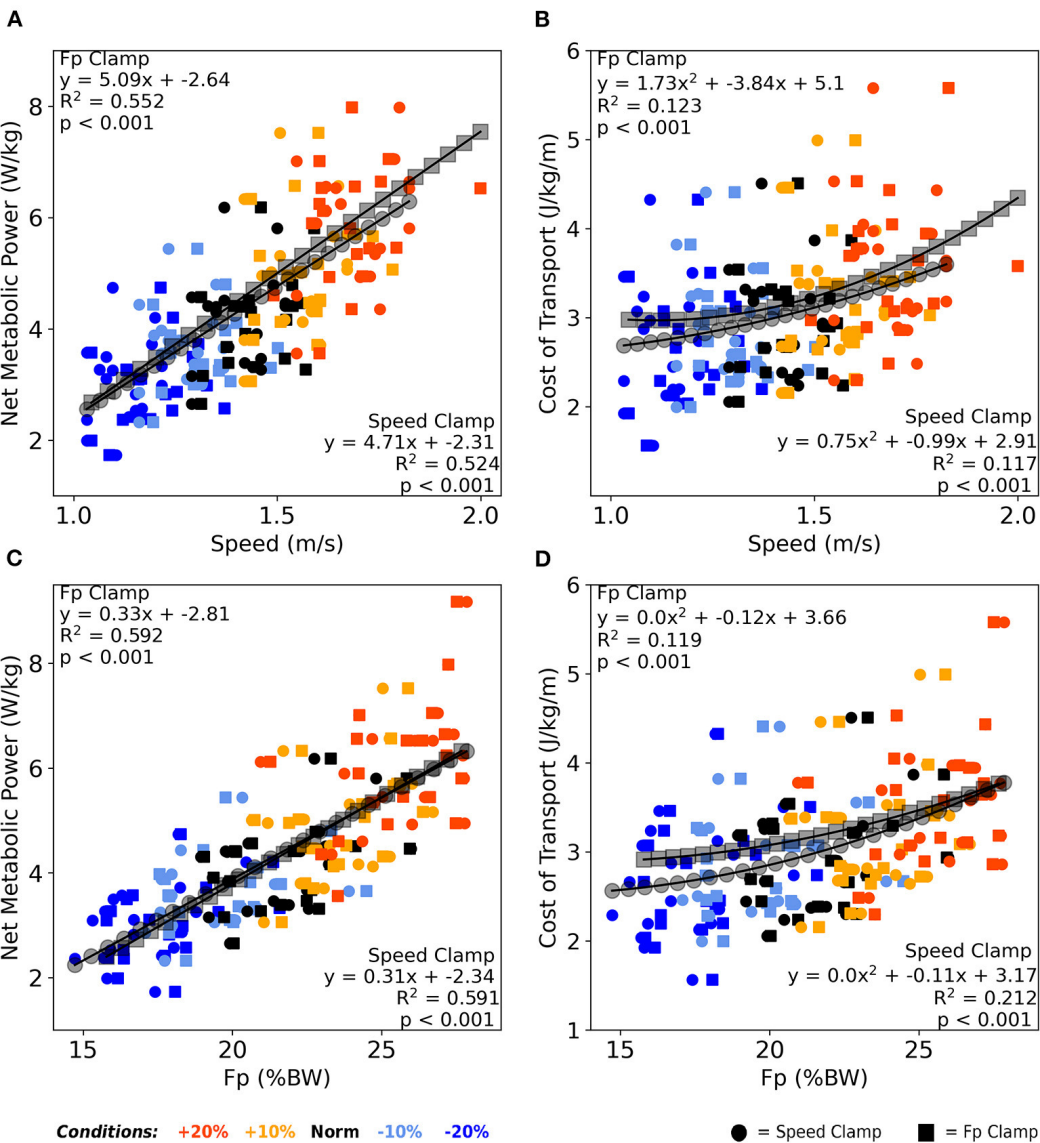
Averaged over the final 2 min, both walking speed **(A)** and F_P_ **(C)** correlated moderately with net metabolic power across both clamp types. Similarly, both walking speed **(B)** and F_P_ **(D)** correlated very weakly with cost of transport across both clamp types. Once again, participants responded nearly identically between clamp types across these four relations as well as between walking speed and F_P_ in the context of metabolic cost. BW, body weight.

Across both clamp types, walking speed moderately correlated with net metabolic power (average *R*^2^ = 0.54, *p* < 0.001, Figure 5A), and very weakly correlated with CoT (average *R*^2^ = 0.12, *p* < 0.001, Figure 5B). Similarly, across both clamps, F_P_ moderately correlated with net metabolic power (average *R*^2^ = 0.59, *p* < 0.001, Figure 5C) and very weakly correlated with CoT (average *R*^2^ = 0.17, *p* < 0.001, Figure 5D).

### Effects of clamp type

**Figure 6:**
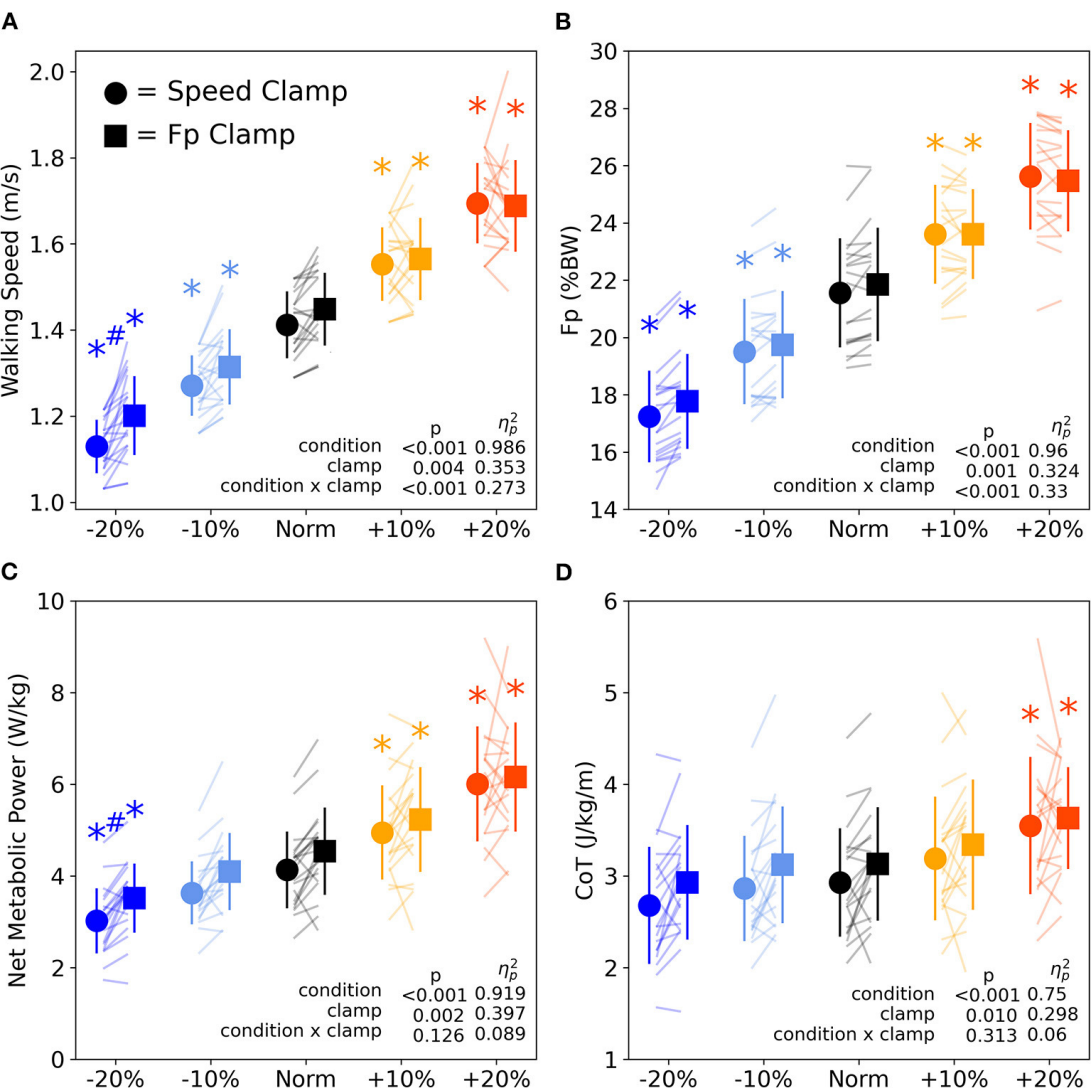
Walking speed **(A)** and F_P_ **(B)** across condition intensities for speed clamp and F_P_ clamp trials. Walking speed and F_P_ similarly increased and decreased with condition intensity. However, at the lowest condition intensity (−20%), particpants walked at faster speeds while extering the same F_P_. Walking net metabolic power **(C)** significantly changed with condition intensity except for the −10% condition intensity. Walking cost of transport **(D)** only significantly increased for the +20% condition intensity. Asterisks (^*^) indicate a significant pairwise *post-hoc* difference (*p* < 0.05) from the Norm condition. Hashtags (#) indicate a significant pairwise *post-hoc* difference (*p* < 0.05) between clamp types (speed vs. F_P_). BW, body weight.

We found significant main effects of clamp type, where, on average across all condition intensities, the F_P_ clamp elicited 2.6% faster speeds, 1.4% greater F_P_ magnitude, 8.9% higher net metabolic power, and 6.2% greater CoT compared to the speed clamp (Figures 6A–D, *p* ≤ 0.01, ηp^2^ ≥ 0.298). We also found significant interactions between condition intensity and clamp type for walking speed and F_P_. The interactions revealed that the difference between clamp types became larger with slower speed and with smaller F_P_ (Figures 6A,B). At the lowest condition intensity, participants walked faster (0.07 ± 0.03 m/s*, p* = 0.008, *d* = 0.887) and with higher net metabolic power (0.49 ± 0.24 W/kg, *p* = 0.044, *d* = 0.660) during F_P_ clamp trails compared to speed clamp trials, despite exerting indistinguishable F_P_ magnitudes.

### Effects of condition intensity

We found significant main effects of condition intensity for all primary outcome variables (F_P_, walking speed, net metabolic power, and CoT, Figure 6). Participants increased and decreased their F_P_ and walking speed in response to higher and lower condition intensities, with each level significantly different from Norm across both clamp types (*p* ≤ 0.002, *d* ≥ 0.957, Figures 6A,B). Net metabolic power increased and decreased along with changes in intensity for all conditions (*p* ≤ 0.049, *d* ≥ 0.642) except when prescribing the −10% condition intensity for both clamp types (*p* ≥ 0.050, *d* ≤ 0.639, Figure 6C). Conversely, CoT only significantly increased from Norm when prescribing +20% condition intensity for both speed and F_P_ clamps (Figure 6D, *p* ≤ 0.013, *d* ≥ 0.827).

## Discussion

Our goal was to objectively investigate relations between F_P_, walking speed, and metabolism across a range of intensities through a unique experimental paradigm designed to separately prescribe F_P_ and walking speed in healthy young adults. This protocol clamped (i.e., held steady) either F_P_ or walking speed, while measuring naturally emergent changes in the other, and recording effects on walking metabolism. Our group and others have observed indirect evidence alluding to interdependency between F_P_ and walking speed and have reported metabolic consequences when walking differs from preferred speed or F_P_ (Hsiao et al., 2015a,b, 2016; Browne and Franz, 2017a, 2018; Conway et al., 2018; Lewek et al., 2018; Conway and Franz, 2020; Hedrick et al., 2021). The recent proliferation of self-pace treadmill controllers (Feasel et al., 2011; Ibala et al., 2019; Castano and Huang, 2021; Hedrick et al., 2021) provides a way to build upon these observational studies. Solidifying the intuition established by prior work (Hsiao et al., 2015a,b, 2016; Browne and Franz, 2017a, 2018; Conway et al., 2018; Lewek et al., 2018; Conway and Franz, 2020; Hedrick et al., 2021), we found strong correlations between F_P_ and walking speed, with ~80% of variance in walking speed explained by F_P_ across both clamp types. Thus, although it need not have been the case, walking with a smaller/larger F_P_ demonstrably led to slower/faster walking speeds, respectively.

Our evidence supporting interdependency between F_P_ and walking speed provides valuable validation for individuals seeking to design and implement strategies to improve walking speed among older adults or individuals with gait pathology. Although walking speed is ultimately governed by interlimb coordination between F_P_ and F_B_ (Supplementary Figures 1, 2), F_P_ has received much more attention as a contributor to walking performance (Peterson et al., 2011; Hsiao et al., 2016; Browne and Franz, 2018; Hedrick et al., 2021; Herrero et al., 2021) and a success metric following intervention (Bowden et al., 2006; Hsiao et al., 2015a; Lewek et al., 2018; Liu et al., 2021). For example, older adults and people with gait pathology walk at slower speeds and with diminished F_P_. Although reduced F_P_ has been implicated as a potential cause of slower walking speeds, their simultaneous presentation makes mechanistic insight difficult. With our documentation of the strong relation between F_P_ and walking speed, we can legitimately identify F_P_ generation as one reason for slower walking speeds. We suspect these strong relations would persist irrespective of the specific mechanism(s) giving rise to diminished push-off in walking. Our results build confidence that interventions designed to augment F_P_ can be used to increase walking speed, as demonstrated by our prior research (Browne and Franz, 2017b, 2019) and relevant work from others (Campanini and Merlo, 2009; Peterson et al., 2011; Hsiao et al., 2016; Hedrick et al., 2021).

### Biomechanics in the context of walking metabolism

Our secondary goal was to establish the metabolic consequences of the interplay between F_P_ and walking speed. We found two key outcomes regarding walking metabolism. Our first key outcome was that net metabolic power was moderately associated with changes in walking speed and F_P_ (*R*^2^ ≈ 0.53, Figures 5A,C) but only weakly associated with CoT (*R*^2^ ≈ 0.14, Figures 5B,D), at least across prescribed changes of ±20% in condition intensity. Previously, our group found that when walking at fixed speeds, both net metabolic power and CoT (inferred from equivalent speeds) increased by ~20% when targeting 20% larger F_P_ (Pieper et al., 2021). By comparison, our current study found that net metabolic power increased by ~40% and CoT increased by ~16% when subjects could self-select their own walking speed while targeting 20% larger F_P_. However, our current findings differed from past results in more obvious ways when targeting 20% smaller F_P_. Previously, both net metabolic power and CoT increased by ~30% when targeting 20% smaller F_P_ using a fixed-speed protocol (Pieper et al., 2021). Conversely, in the current study, net metabolic power decreased by ~25% on average and CoT did not differ from normal walking values when targeting 20% smaller F_P_.

In summary, study differences in the sensitivity of metabolic cost to smaller F_P_ are fully explained by differences in the experimental protocol—namely, prior work being performed at fixed speeds and the current work using a self-paced treadmill controller. These combined results allude to two different scenarios in which a diminished F_P_ can influence walking metabolic cost in the community. First, if walking speed slows when walking with smaller F_P_ in the manner predicted here, then net metabolic power will simultaneously decrease with relatively little change in CoT. Second, should diminished F_P_ precede the selection of slower speeds, for example as we have suggested may occur due to aging (Franz, 2016), compensatory demand on more proximal leg muscles would systematically increase CoT at that speed (Pimentel et al., 2021).

The relative lack of sensitivity of CoT to changes in condition intensity also tends to agree with the “broad minimum” theory, wherein a range of walking speeds neighboring the local minimum of the CoT curve may share similar metabolic costs (Minetti et al., 2003). For normal- and lower-intensity conditions, our participants adjusted F_P_ or speed while maintaining relatively invariant CoT, and thereby operated within their “broad minimum” CoT. Thus, under certain circumstances, walkers may exploit the interaction between F_P_ and walking speed to preserve or reduce walking CoT. Ultimately, changing F_P_ or walking speed predictably alters net metabolic power but need not impact CoT.

Our second key outcome showed that changing the magnitude of either F_P_ or walking speed yielded relatively similar effects on walking metabolism. In other words, whether we prescribed a change in F_P_ or walking speed, effects on walking metabolic cost were nearly indistinguishable. We noted this also from our correlations (Figure 5), which revealed quantitatively similar *R*^2^ values and regression coefficients as well as qualitatively similar trendlines between clamp types. However, there were still meaningful differences between clamp types. We found a significant main effect of clamp type on metabolic cost; F_P_-clamp trials tended to require 9% higher net metabolic power and 6% higher CoT compared to speed-clamp trials on average (Figures 6C,D). We have previously shown that walking with F_P_ biofeedback at a fixed treadmill speed does not itself exact a metabolic penalty (Pieper et al., 2021). We have several possible explanations for the greater metabolic cost associated with F_P_ clamp trials. First, a cognitive “tax” may be required to adjust one's F_P_ and walking speed in response to targeted biofeedback compared to walking at a fixed speed without engaging with biofeedback. Such a tax may allude to additional cognitive processing and/or neuromuscular costs associated with a shift toward supraspinal control of motor output rather than primarily relying on central pattern generators in the spinal cord. Cognitive loads implemented during a dual-task paradigm did not increase walking metabolism (Zukowski et al., 2017). Maintaining targeted motor output control strategies with step-to-step variation may require additional energy compared to simply walking at a fixed speed without biofeedback or walking while performing cognitive tasks without a motor output. Second, the greater metabolic cost exhibited during self-paced F_P_ clamp trials may arise from periodic acceleration/deceleration of walking speed. Although intuitive, we found no association between walking speed variability and net metabolic power or CoT (Supplementary Figure 5). Finally, clamp conditions may be interpreted as constraints, which may alter the metabolic optimization strategies used between those conditions.

### A potential discrepancy in the speed-F_P_-economy relation

Because F_P_ and walking speed are inextricably linked, we would expect our protocol to yield highly similar biomechanical and metabolic outcomes across both clamp types. This was true for most outcomes across most condition intensities (Figure 6). However, when targeting 20% smaller than normal F_P_, our participants could have selected a slower walking speed and lower net metabolic power when producing the requisite F_P_, but they chose not to. Rather, we identified a naturally-emergent discrepancy between clamp types, in which participants exerted similar F_P_, but selected faster speeds at higher net metabolic power during the F_P_ clamp than the speed clamp. The instinctive selection of faster speeds at a metabolic penalty despite indistinguishable F_P_ demonstrates that humans do not *always* seek to minimize metabolic cost (Minetti et al., 2003; Hunter et al., 2010). In our daily lives, we may prioritize factors other than walking economy when we rush, become excited, or feel threatened or scared. We can see evidence of this even in laboratory environments. For example, healthy young adults sometimes select walking speeds somewhat faster than their most economical speed, even though it requires more energy (Minetti et al., 2003). In another example, young healthy subjects have been shown to prioritize stability rather than take advantage of gravity-aided propulsion when walking down a gentle slope (Hunter et al., 2010). These phenomena may explain the discrepancy we identified at low condition intensities, potentially optimizing a cost function other than the most economical gait patterns. We plan to further investigate how participants regulate speed on a step-to-step basis when in self-pace mode, and how lower extremity muscles generate and regulate F_P_.

### Translational implications

It is unclear whether these direct relations between F_P_, walking speed, and walking metabolism will hold in populations who may be candidates for clinical countermeasures to enhance gait performance or mitigate walking-related fatigue. For example, older adults typically walk slower, with smaller F_P_, and at higher metabolic costs compared to young adults. It is actually not well-known whether or not older adults select movement biomechanics and muscle actions that are tuned to minimize metabolic cost at their preferred speeds. Older adults also have the capacity to generate larger F_P_, comparable to F_P_ in younger adults, but typically choose not to utilize that additional force capacity to increase walking speed (Conway and Franz, 2020). Future studies may consider enrolling older adults in a similar design to determine whether age influences the relation between F_P_ and walking speed, or if the metabolic consequences of that relation are altered by hallmark changes in muscle morphology/composition, cardiopulmonary function, sensorimotor integration, or executive processing.

### Limitations

Unique from traditional observational studies, our novel biofeedback design is an important step toward objectively quantifying relations between F_P_ and walking speed. Nevertheless, our interpretations are still based on correlation analyses subject to some limitations. We recognize that the direct relation between F_P_ and walking speed may not hold true in all walking situations or across differing populations. However, we suspect that results apply well across a broad range of steady-state walking situations, absent of other environmental constraints or adversity, such as unstable surface, loss of balance, obstacle avoidance, turning, etc. We contend that results here from self-paced walking have ecological validity with relevance to walking situations outside the lab. However, we understand that laboratory-based protocols have inherent limitations in their ability to emulate real-world behavior. We also acknowledge that, although our results suggest a stronger relation between F_P_ and walking speed than between F_B_ and walking speed, future studies conversely targeting F_B_ using biofeedback may be warranted to fairly identify the relations between F_B_, F_P_, walking speed and metabolism. Another limitation is that our F_P_ magnitudes and walking speeds were limited to a relatively small range compared to other studies that quantify walking metabolism. Our condition intensities deviated 20% from typical gait, yielding speeds between 1 and 2 m/s. Our protocol was informed by the magnitude of changes we would deem clinically meaningful. However, speed-dependent increases in CoT in otherwise healthy young adults do not typically arise until ≤ 1 m/s (Mian et al., 2006; Ortega and Farley, 2007). Another limitation is that we averaged profiles over the final 2 min of each 5-min walking trial. Although subjects had an exploration period and reported comfort with the protocol, further practice could influence walking metabolic cost. Finally, we did not quantify individual determinants of F_P_ such as trailing limb angle and peak ankle moment (Hsiao et al., 2015b; Lewek and Sawicki, 2019).

## Conclusion

Using a unique clamp protocol, we provide empirical evidence that increasing or decreasing F_P_ yields faster or slower walking speeds, respectively. Changing either F_P_ or walking speed also elicits uniform changes in walking metabolic cost, where metabolic power moderately and linearly associated with F_P_ and walking speed. As one important takeaway from this study, our results build confidence that interventions designed to augment F_P_ will likely increase walking speed.

## Data availability statement

The original contributions presented in the study are included in the article, Supplementary material, and linked Github pages (see methods). Further inquiries can be directed to the corresponding author/s.

## Ethics statement

The studies involving human participants were reviewed and approved by Office of Human Research Ethics at the University of North Carolina at Chapel Hill. The participants provided their written informed consent to participate in this study.

## Author contributions

REP: conceptualization, data curation, formal analysis, investigation, methodology, resources, software, validation, visualization, and writing—original draft. JNF: data curation, investigation, and writing—review and editing. MDL: conceptualization, methodology, and writing—review and editing. JRF: conceptualization, methodology, funding acquisition, project administration, supervision, resources, software, and writing—original draft. All authors contributed to the article and approved the submitted version.

## Funding

National Institutes of Health grant R01AG058615 provided funding for this study.

## Conflict of interest

The authors declare that the research was conducted in the absence of any commercial or financial relationships that could be construed as a potential conflict of interest.

## Publisher's note

All claims expressed in this article are solely those of the authors and do not necessarily represent those of their affiliated organizations, or those of the publisher, the editors and the reviewers. Any product that may be evaluated in this article, or claim that may be made by its manufacturer, is not guaranteed or endorsed by the publisher.
