# Supplementary figures and images for: Quantifying mechanical and metabolic interdependence between speed and propulsive force during walking
Source: Front Sports Act Living. 2022 Sep 9;4:942498. doi: 10.3389/fspor.2022.942498 (PMC9500214; doi:10.3389/fspor.2022.942498)

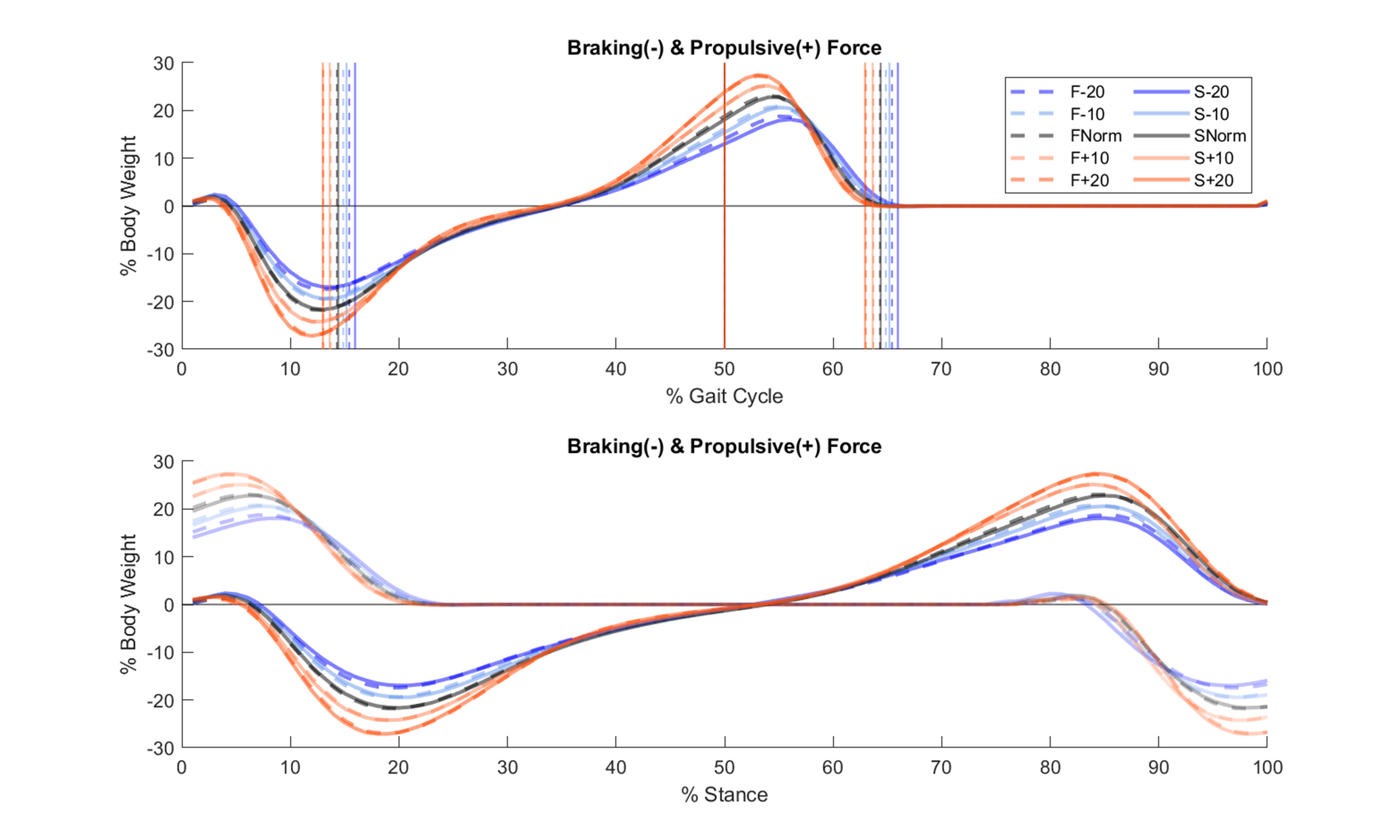

Supplement: Supplementary file 2 [file Image_2.TIF]

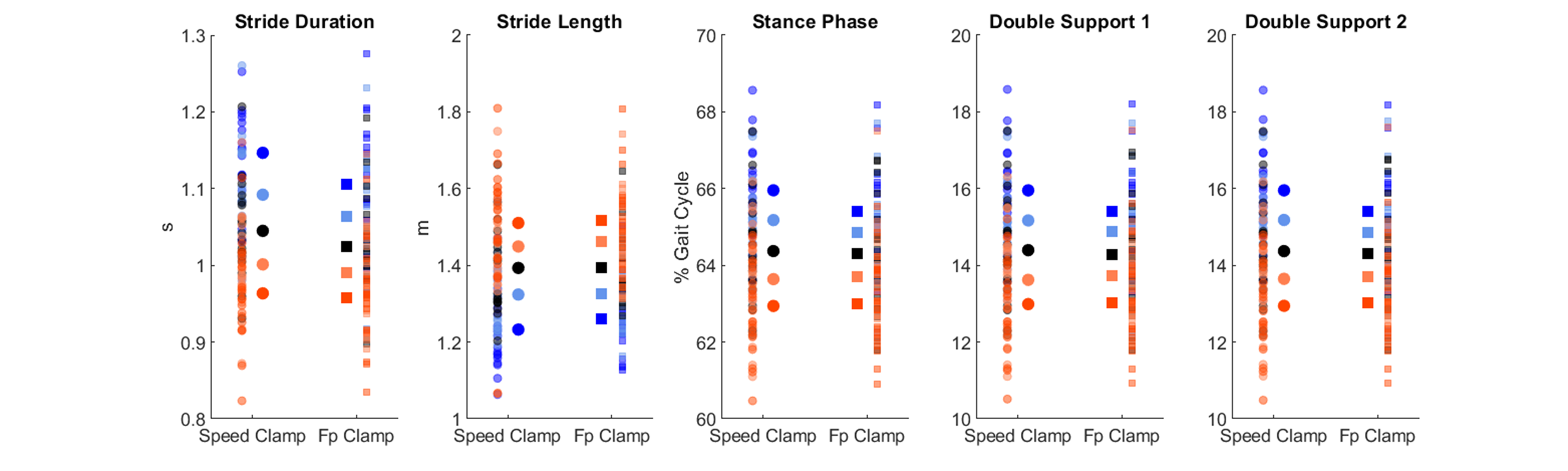

Supplement: Supplementary file 4 [file Image_4.TIF]

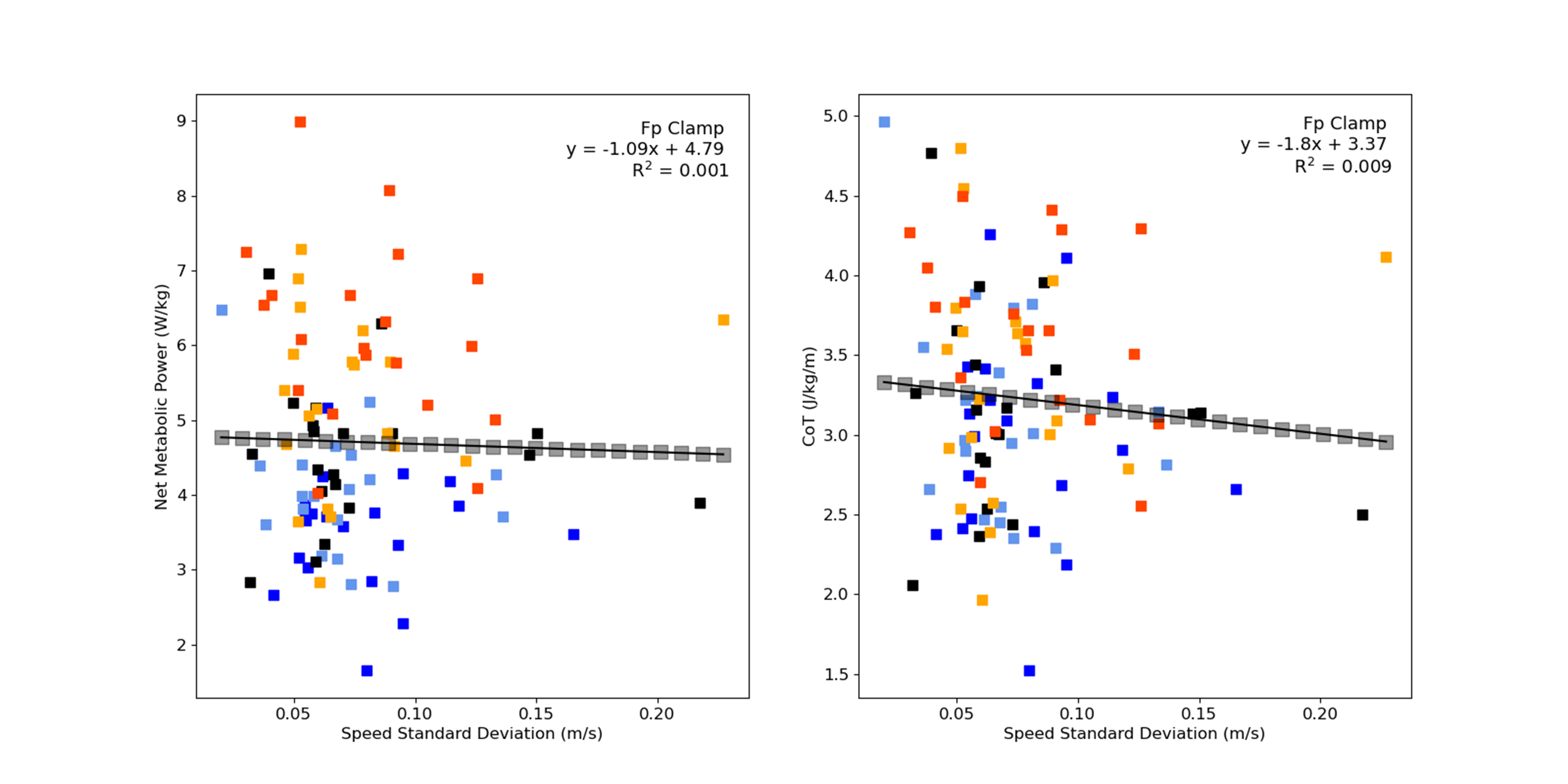

Supplement: Supplementary file 5 [file Image_5.TIF]
